# Nonadherence in Home-Based Pulmonary Rehabilitation Program for COPD Patients

**DOI:** 10.1155/2020/5146765

**Published:** 2020-01-07

**Authors:** Yi Li, Hongyu Qian, Kewei Yu, Ying Huang

**Affiliations:** Department of Respiratory and Critical Care Medicine, Tianjin Chest Hospital, Tianjin, China

## Abstract

**Background:**

The pulmonary rehabilitation (PR) is beneficial for COPD patients. Due to the poor rate of adherence, we evaluate the factors which will predict the nonadherence of PR.

**Method:**

We analyzed the data from a retrospective study of COPD patients who were enrolled to attend the PR program. Patients were classified as the adherence group and the nonadherence group according to completion of over 50% sessions during the 8-week PR program. Demographic characteristics, 6-minute walking distance (6MWD), COPD assessment test (CAT), modified Medical Research Council scale (mMRC), and emotional function were compared between two groups. Univariate and multivariable analyses were performed to determine the factors of poor adherence of PR.

**Results:**

Among 418 patients, 170 patients (40.7%) who completed less than 50% sessions of the PR program were categorized as “nonadherence.” Compared to completers, “nonadherence” patients had more cigarette consumption, higher emotional score, less 6MWD, more exacerbation, using nebulizer frequently, and higher rate of smoking at enrollment. On multivariate analysis, more exacerbation frequency (odds ratio (OR) = 1.434, 95% confidence interval (CI): 1.191∼1.796, *P*=0.046) and smoking at enrollment (OR = 3.349, 95% CI: 1.194∼6.302, *P*=0.012) were predict factors associated with nonadherence of PR.

**Conclusion:**

COPD patients with frequent exacerbation and smoking currently were more likely to be nonadherence during PR.

## 1. Introduction

As the respiratory disease, chronic obstructive pulmonary disease (COPD) has become the global challenge for public health due to its high prevalence and significant mortality [1]. According to the report of Global Burden of Disease Study, COPD is the third leading cause of death in China [2]. Accumulated evidence indicated that the acute exacerbation of COPD triggered by virus and bacterial infection of respiratory tract [3, 4], as well as air pollution [5], increases the cost of health-care resources. To reduce the burden of the disease, the multiple strategies have been recommended in the guideline [6] including pharmacology and nonpharmacology therapies. Pulmonary rehabilitation, a multidisciplinary package of care, which consists principally of exercise training and education sessions as well as self-management, plays an important role in nonpharmacology therapy for COPD patients. Although the benefit of PR was well documented in previous studies [7, 8] showing positive effect on dyspnea, exercise capacity, emotional condition, and health-related quality of life (HQOL), the adherence of the PR program is very poor [9]. The unsatisfied PR adherence will weaken PR benefit and waste the public health resource providing limited value for patients as well as researchers. Previous studies have illustrated that many problems have relation with the adherence of PR among eligible COPD patients. For example, transportation problem [10, 11] is the primary obligation for COPD patients to participate in the PR program; patients who show no concern or lack of motivation with the PR program also have great chance to quit or not to take part in PR from beginning [12]. Furthermore, moderate or severe COPD patients fear that their health would be worsen during the PR exercise. Additionally, socioeconomic and psychological factors, even comorbidities, would affect the adherence of PR [13, 14]. While, the factors associated with adherence of PR were mainly based on the data deriving from outpatients or hospitalization. Recently, the home-based PR maintain research via telecontact provided similar benefit compared to outpatient PR and documented that the adherence of this new strategy is nearly 93% which is much higher than the traditional PR program [15]. The difference of adherence between two forms of PR may be attributed to the superiorities of transportation and easy performing at home. Despite the home-based PR has been recommended as an alternative for COPD patients in the recent guideline [16], the number of studies referred to home-based PR adherence is still limited; furthermore, the conclusion of home-based PR adherence varied greatly regarding to population, socioeconomy, type and duration of the study, and even measurement of adherence.

The purpose of our retrospective study is to compare the demographic and clinical characteristics of COPD patients between the adherence group and the nonadherence group in the home-based PR program and determine the factors which affect the nonadherence of PR.

## 2. Method

### 2.1. Study Population

We retrospectively analyzed data over 5 years (January 2013 through December 2017) from the Respiratory and Critical Care Medicine Department of Tianjin Chest Hospital, which is a tertiary hospital, which offers specialized medical care of pulmonary and cardiovascular disease in Tianjin, China. A total of 484 patients with diagnosis of COPD participated in the 8-week pulmonary rehabilitation program at home within 2 weeks of hospital discharge.

The patient exclusion criteria were as follows: (1) combined with obstructive sleep apnea syndrome (OSAS), based on a combination of patient history (unexplained sleepiness in daytime, snoring, headache during morning, insomnia and concentration difficulty), polysomnography tests, and oxygen saturation monitoring by oximetry [17]. (2) Under diagnosis of cancer. (3) The patients were diagnosed of Alzheimer's or depression and anxious disorders or being suffered from emotional trauma in previous 6 months such as relative death and divorce. (4) Patients were terminated from the program due to acute exacerbation during PR. (5) Incomplete record of PR and questionnaire. This study was approved by the Ethics Committee of Tianjin Chest Hospital. All patients provided written informed consent.

### 2.2. Home-Based Pulmonary Rehabilitation

The home-based PR program was performed under once-a-week phone call interview and self-report diary supervision by the physiotherapist and respiratory nurses in two months. Our PR program includes 3 sessions per week of aerobic training and upper limb resistance training, respectively, and 7 sessions per week of respiratory training; the scheduled total sessions in 8 weeks is 104 (13 ∗ 8). According to the previous study [18], the patients were categorized as “adherence” if they accomplished over 50% sessions; otherwise, they were considered as “poor adherence.”

After the maximum walking speed and maximum heart rate (HR) were identified by using the treadmill before discharge from the hospital, patients were well educated and given general information by the physiotherapist and then started performing tailored PR program or physical training program at home. The exercise program includes (1) individualized aerobic training session with three times per week via outside walking. The targeted HR would be suggested to 75% of the maximum HR [19], starting with 5 minutes and progressively increasing to 20 minutes. (2) Weight-lifting sessions, 0.5 kg-weight dumbbells with 5-second holding for three times every week until peak tolerance. (3) 30-minute respiratory training (half-closed lip abdominal respiratory training and sputum removal training) performing once every day for 2 months. Healthy education was also delivered by respiratory nurses, which emphasizes on the importance of smoking cessation, long-term oxygen therapy, correct usage of respiratory medicine, breathless and emotion management, and nutrition. Meanwhile, recognition of exacerbation, information of the family, and social support will also be provided by respiratory nurses. Furthermore, the respiratory physician will also review the medication regularly.

### 2.3. Measurement

Pulmonary function was completed according to the established standard such as forced expiratory volume in 1 s (FEV_1_), FEV_1_%pred and forced vital capacity (FVC), FEV1/FVC% [20]. Exercise capacity was assessed by six-minute walking test (6MWT) according to the protocol of American Thoracic Society [21]. Health-related quality of life (HRQOL) was evaluated using the COPD Assessment Test (CAT) [22]. Modified Medical Research Council Dyspnea Scale (mMRC) [23] was applied to assess the severity of breathlessness. Beck Depression Inventory (BDI) was used to determine the nervousness, dizziness, and inability to relax. State-Trait Anxiety Inventory (STAI) was used evaluate the severity of the current anxiety and tendency to be anxious [24]. Activities of daily living (ADL) are one of the best tools to evaluate the health condition and the progress of the disease, as well as efficacy of rehabilitation in COPD patients [25].

### 2.4. Statistical Analysis

Data analysis was performed by using Statistical Package for the Social Science (SPSS) version 17.0 software. Continuous data were presented as mean ± standard deviation, and categorical variables were expressed as frequencies (percentages). The chi-square test and independent *t*-test were used to assess the difference between the adherence group and the nonadherence group for categorical and continuous variables, respectively. All variables significantly different between two groups with an alpha of 0.1 were introduced into a multiple logistic regression model to determine the independent predictors of nonadherence. Multivariate odds ratios (ORs) and 95% confidence intervals (CIs) were presented. A probability *P* value of <0.05 was considered statistically significant.

## 3. Results

**Figure 1:**
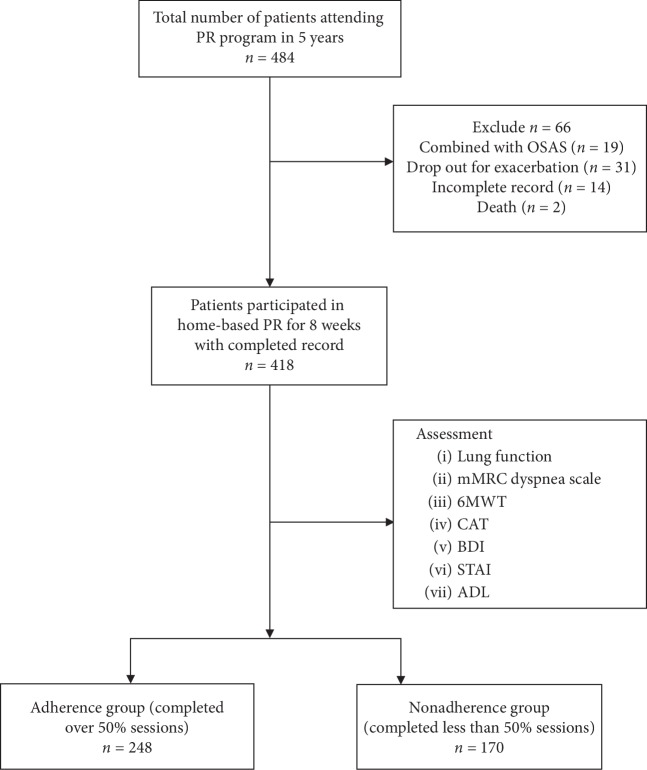
Flowchart of the study population. Note: PR: pulmonary rehabilitation; mMRC: modified Medical Research Council. 6MWT: 6-minute walking test; CAT: COPD assessment test; BDI: Beck Depression Inventory; STAI: State-Trait Anxiety Inventory; ADL: activities of daily living.

A total of 484 eligible patients were recruited in our retrospective study from January 2013 to December 2017, of which 418 patients (86.3%) finished the PR program with completed record. Demographic characteristics of patients at the baseline are shown in Table 1. 248 patients (59.3%) of them completed over 50% sessions, categorized as “adherence,” and 170 patients (40.7%) completed less than 50% sessions (Figure 1). On average, they were older adults (mean age was 65.1 ± 8.5). 297 patients (71%) were former smokers, while 98 patients (23.6%) were current smokers and 23 (5.5) patients who never smoked. The patients in our study had moderate-to-severe COPD (mean FEV_1_predicted is 48.9 ± 12.4%). There were no significant differences between two groups in age, sex, single, BMI, ADL, education, work status, and disease severity (GOLD grade) at the baseline (Table 1). Compared with the adherence group, cigarette consumption (pack-year) was higher in the nonadherence group (28.5 ± 18.3 vs. 36.7 ± 14.5, *P* < 0.001), and the number of current smokers in the nonadherence group at baseline was also higher than the adherence group (17.3% vs. 25.3%, *P*=0.028). Regarding to therapy management, the number of patients who had nebulizer usage is significantly higher in the nonadherence group (35.9% vs. 57.1%, *P* < 0.001), while for nonpharmacological therapy, the usage of noninvasive mechanical ventilator and LTOT were similar in both groups.

There were no significant differences in pulmonary function between two groups (Table 2). As regards six-minute walking distance, the results of 6MWD in the adherence group at baseline was longer than the nonadherence group (340.7 ± 64 m vs. 300.6 ± 33.8 m, *P* < 0.001). However, the results of CAT and mMRC at baseline were similar in both groups with no significant differences. Likewise, there were also no significant differences neither in SAI nor TAI with exception of BDI which is higher in the nonadherence group. Compared with the adherence group, the mean frequency of exacerbation of COPD was higher in the nonadherence group (2.7 ± 1.5 vs. 3.3 ± 1.7, *P*=0.004), while the numbers of hospitalization and emergency visit showed no significant differences in both groups.

On univariate regression, nonadherence was more likely associated with those having more cigarette consumption (OR = 1.008, 95% CI: 1.002∼1.039, *P*=0.021), higher BDI score (OR = 1.072, 95% CI: 1.026∼1.108, *P*=0.013), less 6MWD (OR = 0.749, 95% CI: 0.614∼0.977, *P*=0.037), more frequent exacerbation (OR = 1.409, 95% CI: 1.215∼1.778, *P*=0.002), higher rate of using the nebulizer (OR = 1.066, 95% CI: 1.049∼1.092, *P*=0.029), and higher rate of smoking at enrollment (OR = 3.317, 95% CI: 1.209∼6.288, *P* < 0.001). On multivariate analysis, more exacerbation frequency (OR = 1.434, 95% CI: 1.191∼1.796, *P*=0.046) and smoking at enrollment (OR = 3.349, 95% CI: 1.194∼6.302, *P*=0.012) were predict factors associated with nonadherence of home-based PR (Table 3).

**Figure 2:**
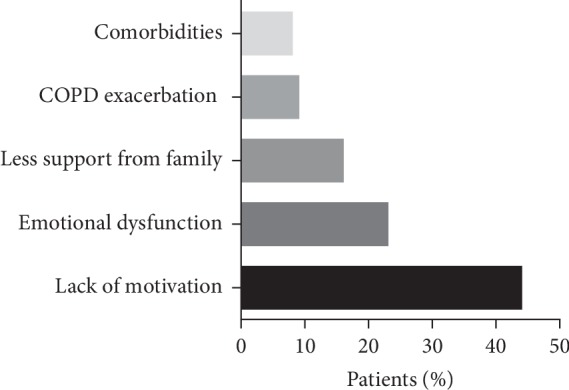
The reasons of nonadherence in home-based pulmonary rehabilitation.

The main reasons of nonadherence concluded by questionnaire survey include five aspects which are as follows (Figure 2): (1) showing no concern about the PR program or lack of motivation, (2) anxiety or excessive focus on their respiratory symptom during the PR program, (3) having less support from family, (4) exacerbation of COPD, and (5) comorbidity. Factors contributed to nonadherence of PR were found to be lack of motivation in 75 patients (44%), anxiety in 39 patients (23%), less support in 28 patients (16%), exacerbation in 15 patients (9%), and comorbidity in 13 patients (8%).

## 4. Discussion

We compared and analyzed the characters of socioeconomic and clinical between adherence and nonadherence groups in our retrospective study which is lasting for 5 years. This study demonstrated that disease severity of COPD dose has significant effect on the adherence of the pulmonary rehabilitation program, though previous research suggested otherwise [10, 11]. In our study, the number of using the nebulizer was higher in the nonadherence group. The 6MWD was also lower in this group. Additionally, compared with the adherence group, the BDI score, an assessment of depression emotion, was significantly higher in the nonadherence group. The frequency of exacerbation of COPD, similar to the clinical characters mentioned above, was higher in the nonadherence group. After adjustment for variables, the predict factors of nonadherence for pulmonary rehabilitation were frequency exacerbation of COPD as well as smoking at enrollment which is similar to the previous study [18]. Furthermore, the leading cause for nonadherence of PR in this study was lack of motivation.

The pulmonary rehabilitation has been shown to be one of the most efficacious nonpharmacological therapies both in stable and postexacerbation COPD patients who relieve the symptom and improve the exercise performance and is recommended by the recent international guideline [1]. Even though, a considerable proportion of COPD patients who discharged from hospital within two weeks failed to participate in PR due to their poor health condition [9]. As severity degree of the disease and the main cause of hospitalization, acute exacerbation of COPD leads to reduction in lung function as well as physical activity, even resulted in worse emotion. Consequently, these patients will more focus on their exacerbation itself rather than pulmonary rehabilitation. Furthermore, they considered themselves to be too ill to complete the program or regarded the pulmonary rehabilitation as so difficult task to perform even postexacerbation. This phenomenon may be so-called “lack of interesting or motivation.” So, we speculate that COPD patients with more frequent exacerbation per year before enrollment have more probability to quit or not to take up this program from initial. This finding was consistent with others [18].

With respect to the second predictor of nonadherence, previous study indicated that smoking increases the nonadherence rate in the rehabilitation program [14, 26]. Similarly, our study demonstrated conclusion that the nonadherence group included much more smokers than the adherence group at recruitment, and after adjustment for variables, cigarette smoking at enrollment is a stronger predictor of nonadherence. Previous research showed that smoking was associated with skeletal muscle dysfunction [27], though this negative effect having relation with nonadherence of PR was not confirmed. However, Young suggested that smoking is a representative of failure management for self-behavior [14]; in other words, those who failed in cigarette cessation during PR will have more great chance to fail in attending or completing the program because they showed no interest or lack of motivation in changing behavior and life style. Furthermore, this unsuccessful behavior management strategy is also related with nonadherence of other rehabilitation programs or pharmacological therapies [28, 29]. Although smoking at enrollment was demonstrated to be a predictor of nonadherence, both ex-smokers and current smokers were still benefited from pulmonary rehabilitation equally when they completed this program as mentioned in the previous study [30]. Given this, we have no more evidence to exclude smokers who did not quit smoking from pulmonary rehabilitation at recruitment. Conversely, cigarette cessation education should be delivered as a crucial part during pulmonary rehabilitation [31] to help them achieve better behavior management. However, the relation between successful cigarette cessation and increased the attending or adherence is still indeterminate.

Besides, factors such as exacerbation and smoking status, other clinical and social demographic characters were also associated with adherence of pulmonary rehabilitation, despite some of these issues were still controversial. Majority of studies suggest that FEV1 value had no effect on PR adherence [12–14]; Sahin and Naz concluded that FEV1 value was lower in patients who fail to complete the PR without multiple logistic regression [32], and Cassidy et al. suggested the FEV1% variable was predictor of completion of PR only with OR 1.01 (95% CI 1.00∼1.02) [26]; however, one study showed that low FEV1 impaired PR program adherence [11]. The patients who live alone were less likely to take up the program due to lack of social support suggested by Hayton and colleagues which is consistent with previous study [12]. Regarding the education level, the same previous study showed that education had no effect on adherence of PR [12]. In contrast to a recent prospective study which demonstrated that patients with lower levels of education would have more likely to drop out or did not attend the program [32], Oates et al. recently indicated that moderate adherence was associated with socioeconomic disadvantage, for example, the income level, unemployment rate, and the number of household vehicles, and the low adherence was associated with limited function [33].

Our study also concluded the main reasons of nonadherence for PR including lack of interest, anxiety, less support, exacerbation, and comorbidity were partially consistent with the previous study with exception of transportation problems [14]. We speculated this phenomenon from advantage of the current PR program which was performed at home overcomes the distance obstacle for patients with longer than 30-minute travel [34]. Though such factors mentioned above were not found to be related with PR adherence in our study, we have no more evidence to ignore their potential significances.

Several limitations of the present study include the following: (1) despite we concluded the predictors for nonadherence in our retrospective study, it is not generalized since this is a single center study with small sample size. (2) Because the clinical and social context are multiple dimensional construct including disease severity, comorbidities, financial, human, social resources, and capital, it is insufficient to identify all domains of socioeconomic and clinical characters being related with adherence of PR. Therefore, we will collect more detail of patients who participate in the PR program and assess more predictors related to adherence of PR in the future multicenter study. (3) Although the method for adherence categorization in our study is simple and convenient to perform, it is insufficient to assess the adherence comprehensively. Therefore, we will evaluate adherence of PR more quantitatively in future study referring to previous research [35, 36].

In conclusion, smoking status and exacerbation of COPD were predictors of PR nonadherence in our retrospective study. Furthermore, as an important component of PR, cigarette cessation should be more concerned and well informed to patients with COPD attending the PR program. Meanwhile, to improve adherence and reduce the wasting of limited financial resources, pulmonary rehabilitation therapists should also ensure the information of patients including clinical and socioeconomic features before PR.

## Figures and Tables

**Table 1:** Demographic characteristics of participants at baseline (*n* = 418).

Variables	All patients	Adherence group (*n* = 248)	Nonadherence group (*n* = 170)	*P* value
Age (years)	65.1 ± 8.5	65.3 ± 8.8	64.8 ± 8.6	0.503
Sex, male, *n* (%)	354 (84.7)	208 (83.9)	146 (85.9)	0.338
Single, yes, *n* (%)	40 (9.6)	22 (8.9)	18 (10.6)	0.336
Work status (still work) *n* (%)	179 (42.8)	101 (40.7)	78 (45.9)	0.172
BMI (kg/m^2^)	23.1 ± 2.7	23.7 ± 2.2	22.6 ± 3.4	0.981
ADLs	16.9 ± 3.6	17.2 ± 3.7	16.4 ± 3.5	0.395
Education (years)	8.3 ± 2.9	8.6 ± 2.7	8.2 ± 3.2	0.494
Smoking (pack-year)	33.8 ± 17.5	28.5 ± 18.3	36.7 ± 14.5	<0.001^*∗*^
Smoking status (*n*)				
Ex-smoker	297 (71)	190 (76.6)	119 (70)	0.091
Smoker	98 (23.6)	43 (17.3)	45 (25.3)	0.028^*∗*^
None	23 (5.5)	15 (6.1)	8 (4.7)	0.129
Comorbidities, *n* (%)				
≥1	369 (88.3)	224 (90.3)	145 (85.3)	0.124
None	49 (11.7)	24 (9.7)	25 (14.7)	
Pharmacological therapy				
Nebulizer	186 (44.5)	89 (35.9)	97 (57.1)	<0.001^*∗*^
None	232 (55.5)	159 (64.1)	73 (42.9)	
Nonpharmacological therapy				
LTOT	185 (44.3)	112 (45.2)	73 (42.9)	0.689
NIV	133 (31.8)	75 (30.2)	58 (34.1)	0.454
Both	71 (17)	44 (17.7)	27 (15.9)	0.691
None	29 (6.9)	17 (6.9)	12 (7.1)	0.542
GOLD grade				
II (50% ≤ FEV1% <80%)	110 (26.3)	67 (27)	43 (25.3)	0.695
III (30% ≤ FEV1% 50%)	219 (52.4)	129 (52)	90 (52.9)	0.852
IV (FEV1% <30%)	89 (21.3)	52 (21)	37 (21.8)	0.845

*Note*. BMI: body mass index; ADL: activities of daily living; LTOT: long-term oxygen therapy; and NIV: noninvasive ventilation. GOLD: Global Initiative for Chronic Obstructive Lung Disease. For categorical variables, the results are expressed as number (percentage); for continuous variables, the results are expressed as mean ± standard deviation. A *P* value less than 0.05 is considered statistically significant and indicated by an asterisk (^*∗*^).

**Table 2:** Clinical characteristics of participants at baseline (*n* = 418).

Variables	All patients	Adherence group	Nonadherence group	*P* value
FEV1 (L)	1.12 ± 0.39	1.13 ± 0.35	1.11 ± 0.42	0.072
FEV1%	48.9 ± 12.4	48.3 ± 12	49.7 ± 13	0.243
FVC (L)	2.36 ± 0.56	2.35 ± 0.5	2.38 ± 0.63	0.108
FEV1/FVC%	47.9 ± 10.7	48.9 ± 10.3	46.4 ± 11.1	0.751
6MWD	324.3 ± 57.7	340.7 ± 64	300.6 ± 33.8	<0.001^*∗*^
CAT	20.2 ± 7.7	19.6 ± 7.9	20.8 ± 7.3	0. 155
mMRC	2.4 ± 0.9	2.16 ± 0.9	2.69 ± 0.8	0.146
BDI	9.6 ± 5.4	8.3 ± 4.9	11.8 ± 5.4	0.021^*∗*^
SAI	38.5 ± 10.6	39.2 ± 10.4	38 ± 10.8	0.340
TAI	43.5 ± 10.7	43.8 ± 11	43.2 ± 10.6	0.363
Exacerbation	2.9 ± 1.6	2.7 ± 1.5	3.3 ± 1.7	0.004^*∗*^
Hospitalization	2.2 ± 1.4	2.3 ± 1.4	2.1 ± 1.6	0.374
Emergency visit	1.8 ± 0.9	1.7 ± 1.0	1.9 ± 1.2	0.280

*Note*. FVC: forced vital capacity (FVC) and FEV1: forced expiratory volume in 1 s. 6MWD: 6-minute walking distance; CAT, COPD assessment test; mMRC, modified Medical Research Council scale; BDI, Beck Depression Inventory; SAI, State Anxiety Inventory; and TAI, Trait Anxiety Inventory. For continuous variables, the results are expressed as mean ± standard deviation. A *P* value less than 0.05 is considered statistically significant and indicated by an asterisk (^*∗*^).

**Table 3:** Univariate and multivariate analyses of independent factors of nonadherence of home-based PR.

Variables	Unadjusted			Adjusted		
	OR	95% CI	*P* value	OR	95% CI	*P* value
Smoking consumption	1.008	1.002∼1.039	**0.021 ** ^*∗*^	1.022	0.977∼1.133	0.201
BDI	1.072	1.026∼1.108	**0.013 ** ^*∗*^	1.081	0.992∼1.230	0.192
FEV1	0.831	0.655∼1.047	0.411	0.901	0.596∼1.118	0.636
6MWD	0.749	0.614∼0.977	**0.037 ** ^*∗*^	0.822	0.589∼1.010	0.155
Exacerbation	1.409	1.215∼1.778	**0.002 ** ^*∗*^	1.434	1.191∼1.796	**0.046 ** ^*∗*^
Pharmacological therapy						
Nebulizer	1.066	1.049∼1.092	**0.029 ** ^*∗*^	1.081	0.940∼1.103	0.187
Smoking status						
Smoker	3.317	1.209∼6.288	**<0.001 ** ^*∗*^	3.349	1.194∼6.302	**0.012 ** ^*∗*^

*Note*. BDI: Beck Depression Inventory; FEV1: forced expiratory volume in 1 s. 6MWD: 6-minute walking distance; OR, odds ratio; CI, confidence interval. A *P* value less than 0.05 is considered statistically significant and indicated by an asterisk (^*∗*^).

## Data Availability

The data used to support the findings of this study are included within the article.
